# Jewel Orchid’s Biology and Physiological Response to Aquaponic Water as a Potential Fertilizer

**DOI:** 10.3390/plants11223181

**Published:** 2022-11-21

**Authors:** Ganka Chaneva, Alexander Tomov, Momchil Paunov, Viktoria Hristova, Valentina Ganeva, Nikolina Mihaylova, Svetoslav Anev, Nikolay Krumov, Zhenya Yordanova, Boris Tsenov, Valya Vassileva, Georgi Bonchev, Miroslava Zhiponova

**Affiliations:** 1 Department of Plant Physiology, Faculty of Biology, Sofia University, 1164 Sofia, Bulgaria; 2 Department of Biophysics and Radiobiology, Faculty of Biology, Sofia University, 1164 Sofia, Bulgaria; 3 Department of Botany, Faculty of Biology, Sofia University, 1164 Sofia, Bulgaria; 4 Department of Immunology, Institute of Microbiology, Bulgarian Academy of Sciences, 1113 Sofia, Bulgaria; 5 Department of Dendrology, Faculty of Forestry, University of Forestry, 1797 Sofia, Bulgaria; 6 Department of Molecular Biology and Genetics, Institute of Plant Physiology and Genetics, Bulgarian Academy of Sciences, 1113 Sofia, Bulgaria

**Keywords:** aquaponics, chlorophyl fluorescence, leaf anatomy, *Ludisia discolor*, plant physiology

## Abstract

*Ludisia discolor* is commonly known as a jewel orchid due to its variegated leaves. Easy maintenance of the orchid allows it to be used as a test system for various fertilizers and nutrient sources, including aquaponic water (AW). First, we applied DNA barcoding to assess the taxonomic identity of this terrestrial orchid and to construct phylogenetic trees. Next, the vegetative organs (leaf, stem, and root) were compared in terms of the level of metabolites (reducing sugars, proteins, anthocyanins, plastid pigments, phenolics, and antioxidant activity) and nutrient elements (carbon, nitrogen, sodium, and potassium), which highlighted the leaves as most functionally active organ. Subsequently, AW was used as a natural source of fish-derived nutrients, and the orchid growth was tested in hydroponics, in irrigated soil, and in an aquaponic system. Plant physiological status was evaluated by analyzing leaf anatomy and measuring chlorophyll content and chlorophyll fluorescence parameters. These results provided evidence of the beneficial effects of AW on the jewel orchid, including increased leaf formation, enhanced chlorophyll content and photosystems’ productivity, and stimulated and prolonged flowering. The information acquired in the present study could be used in addressing additional aspects of the growth and development of the jewel orchid, which is also known for its medicinal value.

## 1. Introduction

*Ludisia discolor* (Ker-Gawl.) A. Rich. is a terrestrial orchid species in the subfamily Orchidoideae [1,2]. It is often described as a “jewel orchid” because of its characteristic leaf coloring, giving high aesthetic value of the plant. An important feature of *L. discolor* is the ability to form symbiotic relationships with mycorrhizal fungi and the production of a wide variety of phytochemical compounds, in particular, plant glycosides, such as kinsenoside, gudieroside, gastrodin, and others [3]. Many of these substances have been studied for their potential pharmacological activity and development of new medicinal products. Application of different fertilizers and other nutrient sources that stimulate plant growth and development could be used in floriculture and in the targeted accumulation of metabolites of interest.

In recent years, studies have been conducted on natural, effective, and low-cost experimental systems, including the recirculating aquaponics with fish wastewater for growing edible plants in attached hydroponic subsystems [4,5]. In return, the removal of nutrients through absorption by plants, in parallel with their microbiota, reduces dissolved ionic substances, which favors fish welfare. The selection of plant species adapted to hydroponic growth in aquaponic systems is related to the fish density in the aquariums and the associated concentration of nutrients in the aquaculture wastewater [4]. Different types of leafy vegetables and plants can be grown in aquaponics; however, due to the specific requirements of individual plant species, preliminary tests and optimizations are needed. Herbs and especially green plants have low to medium nutrient requirements and are well adapted to aquaponic systems [6,7,8]. Several reports describe variability in the biochemical content, size, biomass, and growth efficiency of plants in such systems. However, most of the studies focus on vegetables and herbs, and compare growth between hydroponic and aquaponic cultivation, while research materials on the aquaponic adaptability of many ornamental and rare medicinal plants are, in general, scarce [9,10,11].

The selection of *L. discolor* as a plant species adapted to hydroponic cultivation in aquaponic systems could be a viable approach to improve biomass accumulation and propagation techniques and promote their potential applications. This approach may stimulate the interest of the floriculture and pharmacological industries in such cultivation practices. In this study, the first taxonomic identification of the jewel orchid was performed via DNA barcoding. Biological traits of *L. discolor* were investigated to gain more insight into the physiological mechanisms underlying differential orchid growth in various experimental systems. Plant growth changes and photosynthetic parameters upon cultivation in aquaponic water (AW) in comparison with the water (W)-irrigated control using three different cultivation methods (hydroponic culture, soil, and aquaponic system) were studied.

## 2. Results and Discussion

### 2.1. Taxonomic Identification of L. discolor via DNA Barcoding

We applied DNA barcoding to validate the taxonomic identity of the analyzed *L. discolor*. The sequenced highly conserved nuclear (*ITS*) and chloroplast (*rbcL*, *matK*, *trnH-psbA*) regions after application of barcoding markers, together with homolog sequences retrieved from the database, were used for the construction of phylogenetic trees (Figures S1–S4; [12,13,14,15]). The results showed significant identity with the available *L. discolor* sequences for all DNA barcode markers: 99.87% for *ITS*, 99.27% for *matK*, 98% for *trnH*, and 99.84% for *rbcL*.

### 2.2. Biological Traits of Vegetative Organs of L. discolor Grown in Soil

**Figure 1:**
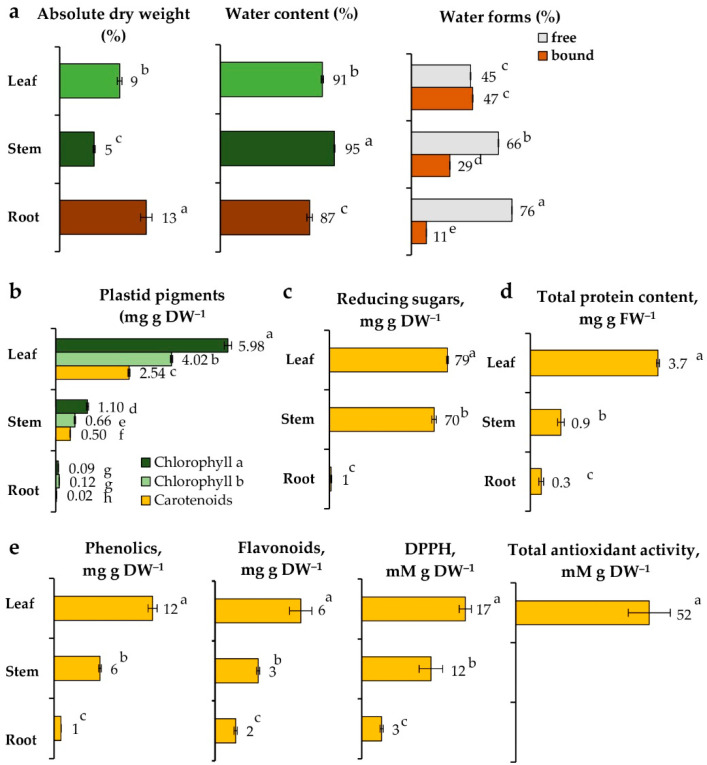
Biological traits of *L. discolor* grown in soil. (**a**) Absolute dry weight, water content, bound and free water; (**b**) Plastid pigments; (**c**) Reducing sugars; (**d**) Total protein; and (**e**) Secondary metabolites and antioxidant activity: total content of phenolic compounds and flavonoids, DPPH anti-radical and total antioxidant activities. Data represent the mean ± SE (*n* ≥ 10). One-way ANOVA (Holm–Sidak) statistical test was applied to estimate the difference among all the variants. Different letters denote statistically significant differences. Scale bar: 5 cm.

To gain more insight into *L. discolor*, we first compared physiological features of leaves, stems, and roots of the orchid plants grown in soil (Figure 1, Figures S5 and S6).

#### 2.2.1. Water Content

Orchid roots accumulated the highest dry biomass (12.6%), followed by the leaves (8.7%) and stems (5.0%) (Figure 1a). Accordingly, water content was relatively high in all organs, with the largest amount detected in stems (95.0%), followed by leaves (91.3%) and roots (87.4%). Similar results for the leaves have been reported by Poobathy et al. [16]. The tropical habitat of *L. discolor* implies the endurance of high temperature and humidity. The obtained results could be explained by the fact that the stem can serve as a water reservoir for the plant. The high bound water in the leaves suggests adaptation against water loss and metabolic activity. The leaves are organs in which many different products of the photosynthesis process, as well as various metabolites, are released, which most likely bind to water [17]. In addition, the presence of stomatal pores in the leaf often results in loss of free water, which is an additional factor contributing to the increased bound water that is rather immobile. The high dry weight of roots could be related to their role as storage organs.

#### 2.2.2. Plant Anatomy

Plants can adjust to variable growth conditions by changing the anatomical structure of different vegetative organs. Hence, we analyzed in detail the anatomy of the *L. discolor* leaves, roots, and stems (Figure S5). Orchids are monocotyledonous plants possessing unifacial or equifacial leaves. Consistent with the previous observations [16], the leaves of *L. discolor* were of the bifacial type, which is atypical for monocots. (Figure S5a). The two epidermal layers had an identical structure. The epidermal cells of the adaxial surface were large, vesicular, and papillary with a thin and smooth cuticle. The cells of the abaxial epidermis were also large and tangentially flattened. The assimilation parenchyma was differentiated into upper palisade parenchyma and lower spongy parenchyma, where the cells were often colored red due to the presence of anthocyanins in the vacuoles. In some of the cells, essential oil drops could be observed. The vascular bundles were collateral and contained typically differentiated phloem and xylem tissues (Figure S5b). The leaves were hypostomatic, having stomata located only on the abaxial leaf epidermis (Figure S5c). The stomata were of an anisocyte type, and each of them was surrounded by three accompanying cells (two large and one smaller). An interesting observation was the presence of idioblastic cells with raphides in the spongy parenchyma parallel to the vascular bundle (Figure S5d). These cells are thought to contain substances that are not used by the plant, but their functional significance has not been elucidated. Raphides are crystals of calcium oxalate that are considered to be involved in the protection against herbivores, such as the monocotyledonous *Pinellia ternata* (Thunb.) Makino, which sharply irritates the mucous membranes of the mouth and laryngopharynx [18]. Similar formations have been observed in members of the genus *Goodyera* and various clusters of orchids [19,20].

The anatomical structure of the two axial organs, stem, and root was not typical (Figure S5e,f). Stems displayed a rhizome-type structure, i.e., a concentric type of vascular bundles, and the roots had not very well differentiated radial polyarchic bundle. The cells of the stem cortex contained chloroplasts (Figure S1e). Inside, the stem parenchymal cells were brighter, and likely, in addition to conducting metabolic processes, they also store water in the vacuoles.

The orchid organ anatomy suggests the ability of *L. discolor* to adapt to water loss (e.g., through the cuticle, stem core, and root hairs) and low and high light intensity (formation of palisade mesophyll with chloroplasts performing photosynthesis, the accumulation of chloroplasts in the outer stem tissue; presence of anthocyanins to scatter excess light). Anthocyanins play a role in thermoregulation and protection against UV light, and it can be assumed that their presence suggests an evolutionary advantage in adapting to environmental conditions [21].

#### 2.2.3. Pigments, Reducing Sugars, Proteins, and Antioxidants

In plants, the photosynthesis process actively takes place primarily in the leaves. The analysis of plastid pigments showed the presence of a high amount of chlorophyll *a* and *b* and carotenoids, which define the first stage of photosynthesis, associated with photochemical activity in thylakoid membranes (Figure 1b). During the subsequent fixation of CO_2_, the sugars are synthesized and transported to other organs of the plant in the phase of active growth or the storage of reserve substances [22]. To determine which organ is enriched in metabolites and would be of potential research interest, we performed a comparative analysis of the metabolic activity of individual *L. discolor* organs, detecting reducing sugars, proteins, phenols, and flavonoids (Figure 1c–e). In support of our hypothesis regarding the bound water, the highest values of primary sugars (79 mg g DW^−1^), proteins (3.7 µg g FW^−1^), and secondary metabolites (12 mg g DW^−1^ phenolics and 6 mg g DW^−1^ flavonoids) were measured in the leaves, where photosynthetic activity occurs. In stems, the content of sugars was close to that in the leaves (70 mg g DW^−1^), while the total protein was four times lower (0.9 µg g FW^−1^), and the secondary phenolic metabolites were reduced by half (6 mg g DW^−1^ phenolics and 3 mg g DW^−1^ flavonoids). In roots, primary metabolites were almost absent (1 mg g DW^−1^ sugars and 0.3 µg g FW^−1^ proteins), and secondary metabolites were less compared with other organs (1 mg g DW^−1^ phenols and 1.5 mg g DW^−1^ flavonoids), as the accumulation of flavonoids might be linked to their transport from other organs to attract microorganisms. Comparison of the protein profiles by SDS-PAGE showed high diversity in the orchid leaves (Figure S6). In stems, the bands were less intensive with increased abundance of a protein with molecular mass of approximately 11–12 kDa. In the roots, less intensive and much weaker bands were observed.

In summary, the orchid leaves showed the highest metabolic activity, followed by stems and roots. These results proved the leaves to be the most suitable organ for further phytochemical studies. Due to the high water content in these organs, a predominant presence of water-soluble compounds with potential biological activities can be expected. Since most phenolic compounds function as antioxidants, we applied two assays to evaluate the antioxidant potential of extracts from the vegetative organs of *L. discolor* (Figure 1e). The radical-scavenging capacity of the extracts and their total antioxidant activity again showed the highest levels in the leaves (17 and 52 mM g DW^−1^, respectively). A previous report correlated the antioxidant properties of the medicinal plant *Teucrium chamaedrys* with the content of phenolic acids, while flavonoids were suggested to be involved in photoprotection and temperature-response regulation [23]. In stems, the antioxidant activity was weaker, and in roots, it was generally absent (Figure 1e). The results indicated that flavonoids are typical for this orchid species, and other studies on the closely related genus *Anoectochilus* support this claim [24]. Flavonoids play an important role in different biological processes, such as photosensitization, energy transfer, action of plant hormones, regulation of respiration and photosynthesis, etc. [25]. It would be of interest to track the secondary metabolites identified for the species and to develop strategies for inducing their accumulation under controlled conditions.

#### 2.2.4. Macro- and Microelements

Further, we compared the distribution of macro- and microelements in *L. discolor* vegetative organs. Differential allocation of the elements in the leaf, stem, and root dry biomass was observed (Table 1). The highest concentration of carbon (C) was detected in the leaves (35.6%), followed by the roots (31.3%) and stems (29.9%), which correlates well with the respective high metabolic activity in the leaves and storage of polysaccharides in the roots. A number of studies have shown that C content varies significantly among plant organs [26,27], which could depend on the environmental conditions and plant species specificity. The nitrogen content (N) was increased in the leaves and stems. This element is transported as nitrate or nitrite from the roots to the photosynthesizing organs, then reduced to ammonia and included in amino acids, proteins, and N-containing compounds [28]. Phosphorus (P) is transported from the root and could be bound in ATP, as well as in specific enzymes [29]. The highest P amount was observed in the stems, followed by the leaves and roots. As a major component of nucleic acids, P also plays a key role in the regulation of protein biosynthesis. In general, individual plant organs can have different requirements for N and P, due to their varying and multiple functions in the plant organism. Potassium (K) is the most abundant and mobile inorganic cation required for the optimal plant growth [30,31]. The largest K concentration was measured in the leaves and stems, but it was almost absent in the roots [31]. The interaction between K and N is an important determinant of plant growth; K can promote N metabolism and enhance the synthesis of amino acids and proteins [32]. Sodium (Na) was also highly represented in the aboveground tissues of *L. discolor*. Sodium distribution in plant organs seems to be species-dependent [33], as this element is not essential for most plants, but it could have beneficial effects, especially under K deficiency. Curiously, the magnesium (Mg), which is a key element of the chlorophyll molecule, had lower levels in the leaves and high concentrations in the stems and roots. The other assessed elements, iron (Fe), manganese (Mn), zinc (Zn), and calcium (Ca), were mainly accumulated in the roots of *L. discolor*, where they seem to be retained for further transportation or stored [34]. The lower concentration of these metal ions in the shoot part compared with the root suggests the ability of plants to control their transport to the stems and leaves, thus protecting them from toxic concentrations.

### 2.3. Aquaponic Water (AW) as a Fertilizer for L. discolor

#### 2.3.1. AW Characteristics

The parameters of AW from aquarium with golden fish were evaluated each week until Week 4 (Table 2). For this period, the pH decreased from 6.42 to 5.35, and the medium conductivity increased nearly threefold from 650 to 1900 µS.m^−1^.

Accordingly, the presence of elements in AW was analyzed at Week 4 (AW ^Week 4^) and compared with that of the soil substrate (Lactofol) of the pot-grown orchid (Table 3).

Compared with the Lactofol substrate, AW ^Week 4^ seemed poorer in elements (Table 3). Nevertheless, according to the experimental setup, the AW was constantly applied to *L. discolor*, and to understand whether it affected the orchid, we further tested plant growth and photosynthetic parameters.

#### 2.3.2. AW Effect on Growth and Photosynthetic Parameters of *L. discolor* Plants

##### AW ^Week 4^ Hydroponics

**Figure 2:**
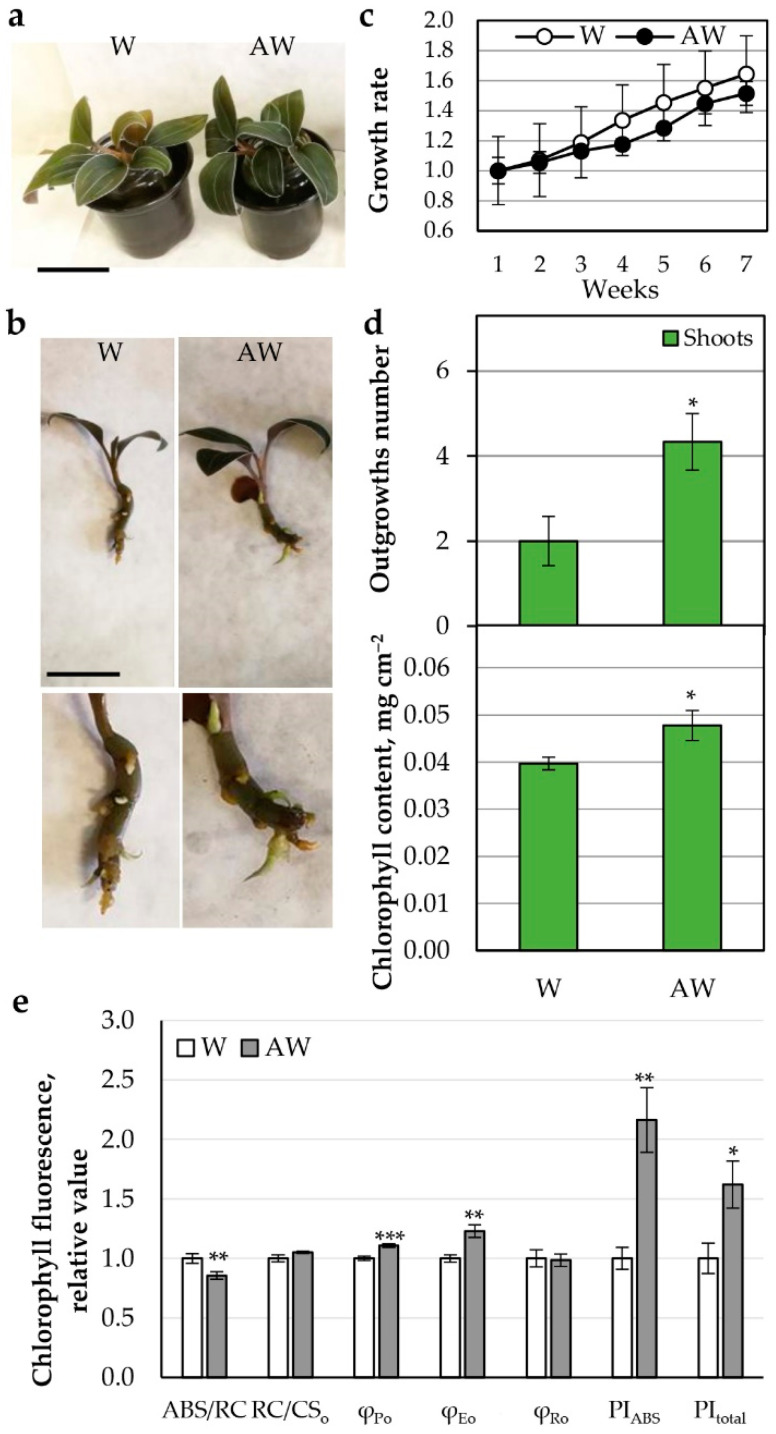
Growth of *L. discolor* hydroponic culture under treatments with AW. The plants were cultivated as hydroponic cultures for 7 weeks. (**a,b**) Photo of the variants: W, water; AW, aquaponic water; Scale bar: 5 cm. (**c**) Growth rate of *L. discolor* fresh biomass for 7 weeks relative to the fresh biomass at Week 1. (**d**) Growth parameters at Week 7: number of outgrowths, chlorophyll content. (**e**) Chlorophyll fluorescence parameters. Statistically significant differences compared with the control W are shown with asterisks after application of Student’s *t*-test (* *p* ≤ 0.05; ** *p* ≤ 0.001; *** *p* ≤ 0.0001).

Soil-grown *L. discolor* was used as a source of orchid outgrowths at similar developmental stage that were placed in parallel in water (W) and in AW ^Week 4^ for hydroponic cultivation. Each week, W and AW were refreshed. For a period of 7 weeks, the growth rate based on the change in fresh biomass was monitored (Figure 2a–c), and the final number of new outgrowths (Figure 2d) at Week 7 was recorded.

In terms of plant biomass, compared with the W control, AW showed no significant change (Figure 2c). Statistically significant stimulation on the formation of shoot outgrowths was observed upon AW cultivation. This result supports the previously described beneficial effect of aquaponic biofertilizer on the plant organism [35].

We followed the effect of treatment with AW fertilizer at the functional level, examining chlorophyll content and photosynthetic performance. In AW, an increase in chlorophyll content was observed (Figure 2d). Analysis of electron transport (from photosystem II/PSII donor, H_2_O, to photosystem I/PSI end acceptor, ferredoxin, and NADP^+^) of the light phase of photosynthesis revealed significant changes in the AW variant (Figure 2e). The relative antenna size of an active PSII reaction center (RC) corresponding to the ABS/RC metric was reduced in AW relative to the control. Since there was no change in the number of active reaction centers per unit area (RC/CS_o_), it could be concluded that the absorbed light energy flux ABS was reduced in AW. The weaker ABS was coupled to more efficient photochemistry and electron transfer from PSII to electron carriers between both photosystems, including plastoquinone (PQ) molecule pool, i.e., increased overall quantum efficiencies (φ_Po_ and φ_Eo_), which correspondingly induced PSII performance index (PI_ABS_). As a result, the total productivity of the whole electron transport chain was higher upon AW hydroponic application than in W control (PI_total_), although electron transfer through PSI did not change its quantum yield (φ_Ro_) per se.

##### AW ^Week 4^ Fertilized Soil

**Figure 3:**
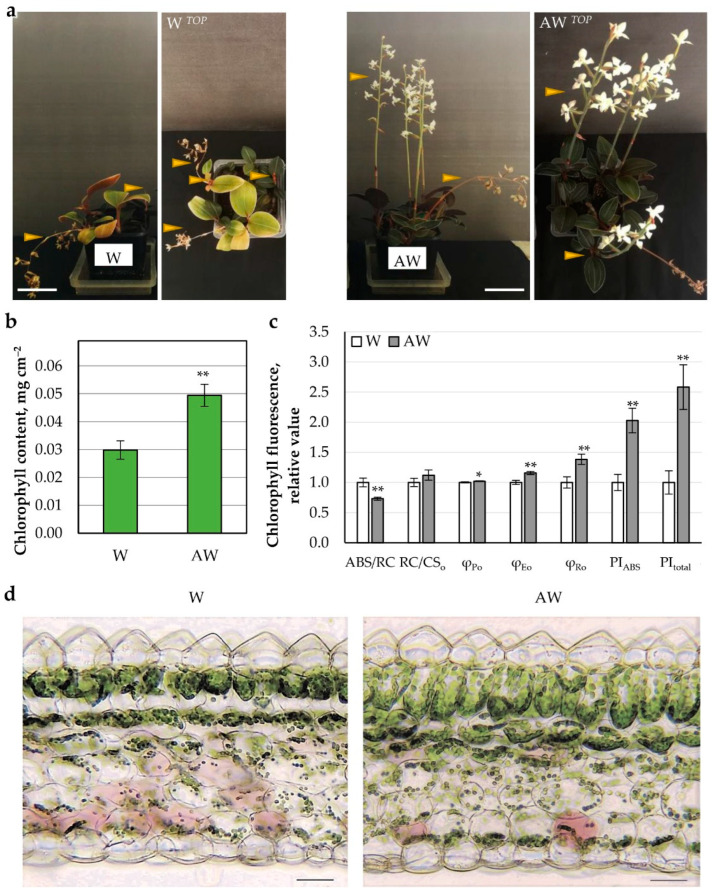
Growth of *L. discolor* in soil under treatments with AW. The plants were cultivated 2 months as hydroponic cultures and transferred in soil for 10 months. (**a**) Photo of the variants: W—water; AW—aquaponics water; Scale bar: 5 cm. Arrowheads point to flower formation. (**b**) Chlorophyll content. (**c**) Chlorophyll fluorescence parameters. (**d**) Leaf anatomical structure of *L. discolor* (transverse sections were made from developed leaves); Scale bar: 50 µm. Statistically significant differences compared with the control W are shown with asterisks after application of Student’s *t*-test (* *p* ≤ 0.05; ** *p* ≤ 0.001).

Similar to the hydroponic culture, in soil-grown orchids watered with AW, the chlorophyll content was increased and the electron transport in PSII and from PSII to PSI was enhanced (φ_Po_ and φ_Eo_) (Figure 3).

Moreover, the PSI displayed even more efficient electron transfer (φ_Ro_), which resulted in much higher overall productivity of the thylakoid photosynthetic machinery (PI_total_) upon AW application (Figure 3b,c). The flowering set up was almost identical in the two variants; however, it was more prolonged upon AW watering (Figure 3a).

The overall leaf anatomical structure was preserved in both W and AW variants (Figure 3d). Detailed observation revealed enhanced thickness of the AW leaf lamina due to enlarged mesophyll (Table 4). In AW, the palisade parenchyma increased by 18%; the palisade cells had longer elongated anticlinal walls and stronger structural contact with each other. The spongy cells were rounded, but smaller in size, and the average thickness of this tissue was 8% higher compared with the control plants. In both variants, the main epidermal cells of the adaxial epidermis were morphologically similar, with the average thickness recorded for plants in AW medium being 9% less. The average thickness of the abaxial epidermis was equal between the variants.

The leaf anatomy study suggested positive effect of AW on the regenerative processes of the leaf at the tissue level, and especially on the structuring of the palisade parenchyma. Subsequent flow cytometric analysis on W and AW leaves did not show clear difference in the DNA duplication, although there was a trend towards increase in endoreduplication in the leaves of the AW variant (Figure S7).

##### AW System (AWS)

**Figure 4:**
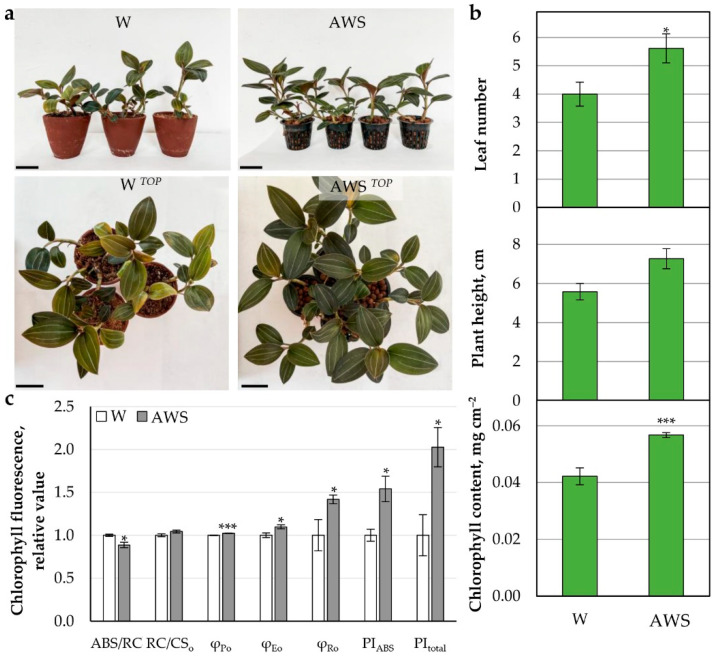
Growth of *L. discolor* in AW system (AWS). The plants were cultivated 2 months as hydroponic cultures and transferred in soil for 2 months and in AWS for 8 months. (**a**) Photo of the variants: W—water irrigation in soil; AWS—aquaponic water system; Scale bar: 5 cm. (**b**) Leaf number per plant, plant height, and chlorophyll content. (**c**) Chlorophyll fluorescence parameters. Statistically significant differences compared with the control W are shown with asterisks after applicationof Student’s *t*-test (* *p* ≤ 0.05; *** *p* ≤ 0.0001).

An aquaponic water system (AWS) was constructed (Figure S8), and orchids at similar developmental stage were placed in parallel in soil irrigated by water (W) as control, and in actively circulating AWS (Figure 4a). The plants adapted successfully to cultivation in AWS, forming more leaves, while the plants’ height remained equal to the W control in soil (Figure 4b). Similar to the treatments described above, the cultivation in AWS positively affected chlorophyll level and photosynthetic electron transport efficiency of both PSII and PSI (PI_total_) in the leaves of *L. discolor* (Figure 4b,c).

Interestingly, increased PSI performance was observed in lettuce cultivated in an aquaponic system despite the decreased PSII activity attributed to the high sensitivity of PSII to Fe- and K-deficient AWS [5]. In this recent study, the concentration of Fe was five times higher than the one presented here, while K levels were comparable. However, the nutrient demand may differ significantly between the two examined species. Correspondingly, the protein level was increased by 21% in AWS, although the protein profile did not differ from that of the W control (Figure S9).

## 3. Conclusions

The leaves of *L. discolor* are the organs with the highest metabolic activity, followed by stems and then by roots. AW was rich in a number of nutrients, including ammonium ions, nitrates, sulphates, and other products of fish excretion, which could potentially have a nourishing and stimulating effect on the orchid. It is tempting to speculate that AW is closer to the soils, where naturally *L. discolor* grows, since the soils in Indonesia are acidic and sulphate-containing [36]. The obtained results indicate that AW, suggested as a potential fertilizer, could be purposely applied for vegetative propagation of *L. discolor*, as it positively affected plant physiological state upon cultivation as simple hydroponic culture, upon soil irrigation, and in actively circulating aquaponic system. In addition, the leaves remained greener, and the flowering was prolonged, which could have floriculture value.

Future research will focus on more detailed identification of AW composition and whether it contains amino acids, peptides, and nucleotides with growth-promoting effect. Another useful tool for studying *L. discolor* could be its cultivation in controlled conditions as in vitro cultures. Applying AW and other fertilizers would provide valuable information for the orchid’s requirements linked to the modulation of its architecture and production of metabolites with pharmaceutical and nutritional importance.

## 4. Materials and Methods

### 4.1. Plant Material and Cultivation Conditions

Ornamentally grown individuals of the species *L. discolor* (Ker-Gawl.) A. Rich. with a completed flowering phase (March) were used as experimental materials. The plants were cultivated in greenhouse conditions under moderate light and temperatures in the range of 20–25 °C and regularly watered when the soil substrate (Lactofol^®^) dried. For treatment with aquaponic water (AW, water from the tank with goldfish *Carassius auratus* L. [4]) and tap water (W) as a control, vegetative outgrowths at the same developmental stage were used. The plants were illuminated with white light at a 16/8 h photoperiod and an intensity of 80 μmol m^−2^ s^−1^. Plants treated with W and AW were cultivated in three different ways: (1) Hydroponic cultivation in light-tight containers containing AW (taken from aquarium with goldfish) or W-irrigated control. Both AW and W were refreshed weekly for 2 months. (2) Pots with Lactofol soil (for plants grown previously as hydroponic cultures for 2 months) that were watered once a week with AW or W for 10 months until flowering. (3) Functional aquaponics system (AWS) equipped with expanded clay pad and PVC siphon (Figure S8; [37]). Equal access to water and oxygen was ensured by repeated cycles between the complete immersion of the plant roots in the circulating water and the complete drainage of the water. Keramzite (lightweight expanded clay aggregate, LECA) was used to fix the plants. Orchid plantlets at similar developmental stage (grown 2 months in hydroponics and 2 months in soil) were placed for 8 months in Lactofol soil irrigated by water (W) as control, and in actively circulating AWS.

### 4.2. DNA Extraction and DNA Barcoding Analysis

Genomic DNA was extracted from *L. discolor* leaves using DNeasy Plant mini kit (Qiagen, Hilden, Germany), according to the manufacturer instructions. The taxonomic identification of *L. discolor* sample was performed through DNA barcoding on the basis of the sequences of four gene regions: nuclear ribosomal internal transcribed spacer (ITS), ribulose-1,5-bisphosphate carboxylase/oxygenase large subunit (*rbcL*) gene, maturase K (*matK*) gene, and *psbA-trnH* intergenic spacer. The primer sequences (synthesized by Microsynth) and PCR conditions that varied among primers are shown in Table S1 [38,39,40,41,42,43]. PCR amplification was performed in 20 μL reaction mixtures containing approximately 30 ng of genomic DNA, 1× PCR buffer, MgCl_2_ (2.0 mM for ITS, 2 mM for *matK*, 2.5 mM for *rbcL*, and *trnH-psbA*), 0.2 mM of each dNTP, 0.2 μM of each primer, and 1.0 U Taq DNA Polymerase (Solis BioDyne, Tartu, Estonia). Amplicon products for all four gene regions were sequenced in both directions by Microsynth (Göttingen, Germany) with the same primers used for PCR amplification. Candidate DNA barcode sequences for each barcode region were edited and aligned in the software package Molecular Evolutionary Genetics Analysis (MEGA) ver. MEGA X [44], and consensus sequences were subjected to further analyses. The consensus sequences for each DNA barcode region are shown in Table S2. Taxonomic assignment of the analyzed sequences was performed using Geneious software via Basic Local Alignment Search Tool (BLAST) matches (http://blast.ncbi.nlm.nih.gov/Blast.cgi, accessed on 23 October 2022) against NCBI nucleotide database. Phylogenetic and molecular evolutionary analyses were conducted using MEGA X software. Phylogenetic trees for each individual barcode region were constructed using the neighbor-joining method [45], and evolutionary distances were calculated based on the best DNA model for each gene alignment. The stability of the topology of the phylogenetic tree was assessed in the bootstrap test (500 replicates).

### 4.3. Physiological Parameters

#### 4.3.1. Water Content

Water content and absolute dry weight (DW) of the tissues were determined after incubation at 105 °C for 5 h. The free water was measured by osmosis in 60% sucrose, and the bound water was calculated by subtracting the free water from the water content.

#### 4.3.2. Histological Analyses

Fresh cross-sections were made through the middle of the blade of fully expanded leaves, through the stem, and through the root and fixed in 3% (*w/v*) glutaraldehyde buffered with 0.1 M sodium phosphate to pH 7.4 for 12 h at 4 °C. Handmade transversal sections (at least 30 per species) were mounted on slides in glycerol. Observations were carried out and micrographs were taken using a light microscope and a camera (Nikon Eclipse 50i, Tokyo, Japan). Leaf anatomy was characterized by measuring the thickness of leaf lamina, palisade parenchyma, spongy parenchyma, and both adaxial and abaxial epidermis. Fifteen measurements for each parameter and treatment were taken (using ImageJ 1.53 K).

For microscopic observations, leaf-clearing from chlorophyll and other pigments was performed by ethanol, 1.25 M NaOH:EtOH (1:1) and lactic acid, as previously described [46]. The cleared leaf samples were placed on a glass slide in lactic acid with the abaxial side (with less trichomes) facing up. The samples were imaged under white light under a microscope (MRC Scientific Instruments), and the pictures were taken with the attached DV-300 camera and LissView program Version 6.1.4.1. The middle part of the leaf between the midrib and the periphery was photographed. The Nikon SMZ 745 binocular microscope was also used for the fresh sections.

#### 4.3.3. Biochemical Analyses

Fresh plant material (leaves, stems, and roots) was frozen in liquid nitrogen and lyophilized for 36 h by lyophilizer Biobase BK-FD10 (Biobase Biodustry Shandong Co., Ltd.). The material was used for the subsequent metabolic analyses.

Pigment analysis was carried out spectrophotometrically, according to a standard procedure described by Arnon [47]. The contents of chlorophyll a, chlorophyll b, and carotenoids were determined after measuring the extinction in a total 80% acetone extract on a Shimadzu UV 1800 spectrophotometer. The concentration of pigments was determined by the absorption coefficients in the formulas proposed by McKinney [48].

The content of reducing sugars was determined via 3,5-dinitrosalicylic acid (2-hydroxy-3,5-dinitrobenzoic acid), according to Plummer [49] and Asare-Brown and Bullock [50]. The content of reducing sugars was measured via a standard curve using glucose as a standard and expressed as mg glucose per g dry weight (mg g DW^−1^).

Total protein was measured according to Kielkopf et al. [51]. Analytical gel electrophoresis, SDS-PAGE, of protein samples was performed on 11% polyacrylamide slab gels [52]. Protein was detected by silver staining, as described by Nesterenko et al. [53]. As size standard, the PageRuler™ Prestained Protein Ladder, 10–170 kDa (Thermo Scientific™) was utilized.

Plant material was used for preparation of crude methanol extracts (10 mg mL^−1^) by homogenization and ultrasonication. The procedures for spectrophotometric quantification of phenolics and flavonoids and antioxidant activities (total antioxidant activity, TAA, and radical-scavenging activity using stable 2,2′-diphenyl-1-picrylhydrazyl, DPPH, free radical) were described by Zhiponova et al. [23] and Petrova et al. [54]. The content of polyphenols was determined according to Singleton et al. [55] by a standard curve created using the known concentrations of gallic acid (GA). The content of flavonoids was measured according to the protocol of Chang [56] by a standard curve based on the known concentrations of quercetin (Q). The total antioxidant activity was assayed by the method of Prieto et al. [57] using α-tocopherol as a standard. The DPPH-radical-scavenging activity was measured using Trolox as a standard, according to the method of Brand-Williams et al. [58] The spectrophotometric measurements were performed with spectrophotometer Shimadzu UV 1800 (Kyoto, Japan).

#### 4.3.4. Plant Growth

The plant growth in hydroponic culture was determined by measuring the fresh biomass of an individual plant over 7 weeks and presenting as a ratio to its initial biomass. On the 7th week, the number of newly formed outgrowths were recorded. In soil-grown plants, the flowering was compared at the 12th month. In control and AWS variants, the leaf number was counted, and plant height was measured by a ruler at the 12th month.

#### 4.3.5. Flow Cytometry

Fresh leaf tissues (as shown on Figure S7a) were chopped with a razor blade in 1 mL Galbraith buffer (45 mM MgCl_2_.6H_2_O; 30 mM Na_3_Citrate.2H_2_O; 20 mM MOPS, pH 7.0; 0.3% Triton-X-100) added consecutively in two portions. The suspension was filtered through a 50 µm mesh, and propidium iodide (50 µg.mL^−1^) was added. The distribution of the nuclear DNA content was analyzed with LSR II (BD Bioscences, Franklin Lakes, NJ, USA) using FlowJo v10 software (FlowJo, Ashland, OR, USA). At least twenty thousand nuclei were analyzed in each sample with three replications [59].

#### 4.3.6. Mineral Element Assay

In plant biomass, C was defined by decomposition and oxidation to CO_2_ in highly acidic medium followed by titration approach. Total N was determined by the Kjeldahl method via heating the samples to 360–410 °C for 30 min in the presence of concentrated H_2_SO_4_, which leads to oxidation and liberation of N as (NH_4_)_2_SO_4_. The samples were subjected to distillation in the presence of NaOH for alkalization and release of NH_4_OH, which was captured in 4% H_3_BO_3_, and the NH_4_^+^ concentration was determined by titration with 0.1 M HCl. In the liquid sample, the concentration of NH_4_^+^ (C_NH4+_) was determined directly after distillation in alkali medium and titration. To determine NO_3_^−^ concentration, in addition to the available NH_4_^+^, NO_3_^−^ were subjected to reducing agent for reduction to NH_4_^+^, followed by distillation and titration (i.e., C_NO3-&NH4+_). The NO_3_^−^ concentration (C_NO3-_) was calculated as the difference between C_NO3-& NH4+_ and C_NH4+_.

For measurement of P and the elements Fe, Mn, Zn, Mg, Ca, K, and Na in plant biomass, dry burn at 450 °C was performed, followed by dissolving in mineral acid (20% HCl), while the liquid AW sample was used directly for analysis. The above elements, except P, were analysed by Atomic absorption spectrophotometer (Perkin Elmer M-5000) and using standards. The measurement of P was done colorimetrically and via standard. The soluble S (SO_4_^2−^) was measured by precipitation with BaCl_2,_ followed by UV measurement and standards.

### 4.4. Chlorophyll Content and Fluorescense

Chlorophyll content in fully developed leaves was assessed by a non-destructive method using a portable chlorophyll meter atLEAF (at-LEAF FT-GREEN-LLC Willington, DE, USA) that estimates the intensity of green color of the leaves at two wavelengths: 653 nm (red color) and 931 nm (infrared color) [60]. Chlorophyll fluorescence was measured as described by Zhiponova et al. [61]. Briefly, chlorophyll fluorescence induction curves were obtained in fully developed leaves in vivo by *M-PEA* fluorimeter (Multi-Function Plant Efficiency Analyser, Hansatech Instruments, King’s Lynn, UK) for 1 s with PAR of 4000 μmol (photons) m^−2^s^−1^ with two recordings per leaf. The whole plants were adapted to darkness for 1 h prior to the measurements, which were conducted in the morning between 10:00 and 12:00 h. The data processing was carried out in M-PEA Data Analyser 5.4 program at first, then seven fluorescence parameters were calculated in Microsoft Excel, according to JIP-test: (1) ABS/RC, light absorption flux (by antenna chlorophyll molecules) per Q_A_-reducing (active) PSII reaction center(s) (RC), a measure of PSII apparent antenna size; (2) RC/CS_o_, area density of active reaction centers, CS_o_, the illuminated cross-section of the leaf; (3) φ_Po_, maximum quantum yield of PSII primary photochemistry (i.e., photooxidation of RC chlorophyll P_680_ and reduction of primary quinone acceptor Q_A_); (4) φ_Eo_, quantum yield for electron transport from PSII to the acceptors between the PSII and PSI (mainly PQ); (5) φ_Ro_, quantum yield for electron transport from PQ to the end electron acceptors at the PSI acceptor side (ferredoxin and NADP^+^); (6) PI_ABS_, performance index for energy conservation from photons absorbed by PSII antennae to the reduction of intersystem electron acceptors; and (7) PI_total_, performance index for energy conservation from photons absorbed by PSII to the reduction of PSI end acceptors.

### 4.5. Statistical Analysis

All measurements included plant material from three biological repeats involving three technical replicates (*n* ≥ 3). Each biological repeat contained plant material from an average of 15 plants (*n* ≥ 15). The results are presented as mean values ± standard errors (SE). To evaluate statistical differences among different organs, one-way ANOVA, followed by Holm–Sidak test was performed using Sigma Plot 11.0 software. The statistical differences between the W and AW treatments were estimated by Student’s *t*-test. The differences were considered significant at *p* < 0.05.

## Figures and Tables

**Table 1:** Composition of elements in vegetative organs of *L. discolor*. Data are expressed per g DW. Representative values are obtained from a pooled sample (6.0 ± 1.0 g DW) of 50 individual plants (*n* ≥ 50).

	^1^	C	N	P	^2^	K	Na	Mg	Fe	Mn	Zn	Ca
Leaf		35.6	2.8	0.5		45.1	3.0	1.4	105	31.5	45.5	2.8
Stem		29.9	2.5	0.8		43.9	6.4	3.7	165	31.5	54.5	7.4
Root		31.3	1.4	0.3		4.4	0.9	2.3	4095	216.8	164.5	11.3

^1^ % (i.e., g 100 g DW^−1^). ^2^ µg g DW^−1^.

**Table 2:** Changes in AW parameters over time.

	Week 0	Week 1	Week 2	Week 3	Week 4
pH	6.42	6.32	5.89	5.71	5.35
Conductivity, µS.m^−1^	650	750	1150	1390	1900

**Table 3:** Composition of elements in AW at Week 4.

**^1^**	**NH_4_^+^**	**NO_3_^−^**	**P_2_O_5_**	**SO_4_^2−^**	**K**	**Na**	**Mg**	**Fe**	**Mn**	**Zn**	**Ca**
AW ^Week 4^	23.43	7.73	12.12	6.11	7.93	2.03	1.33	0.02	0.04	0.26	5.50
Lactofol ^2^	200		150	150	270	n/a ^3^	100	n/a	n/a	n/a	n/a

^1^ mg.L^−1^; ^2^ soil substrate used for comparison; ^3^ n/a, not available information.

**Table 4:** Thickness (μm) of leaf lamina and its tissues and palisade factor (%) in *L. discolor* plants grown in W-irrigated and AW ^Week 4^-fertilized soil.

Parameter	W	AW
Leaf lamina	370.38 ± 2.40	405.34 ** ± 17.84
Mesophyll	265.54 ± 6.78	295.85 * ± 26.59
Palisade parenchyma	76.66 ± 6.52	91.91 * ± 0.99
Spongy parenchyma	197.23 ± 2.29	214.22 * ± 1.28
Adaxial epidermis	75.76 ± 9.79	61.97 * ± 8.60
Abaxial epidermis	57.87 ± 0.13	55.95 ± 13.93

Means ± standard errors (SE) of 15 measurements per variant are shown. Statistical significance difference compared with the control plants were calculated by Student’s *t*-test, asterisks indicate *p* value: * *p* ≤ 0.05, ** *p* ≤ 0.01.

## Data Availability

The sequences for the DNA barcoding markers were deposited in the Genbank database of NCBI under the following accessions: *ITS* OP688578; *rbcL* OP719316; *matK* OP719315; and *trnH-psbA* OP719317.

## Supplementary Materials

The following supporting information can be downloaded at: https://www.mdpi.com/article/10.3390/plants11223181/s1: Figure S1. Phylogenetic tree of *Ludisia* sp. for the ITS DNA barcoding marker; Figure S2. Phylogenetic tree of *Ludisia* sp. for the matK DNA barcoding marker; Figure S3. Phylogenetic tree of *Ludisia* sp. for the *rbcL* DNA barcoding marker; Figure S4. Phylogenetic tree of Ludisia sp. for the *trnH-psbA* DNA barcoding marker; Figure S5. Anatomical overview of vegetative organs of *L. discolor* grown in soil; Figure S6. SDS PAGE protein profiles of *L. discolor* leaf, stem, and root; Figure S7. Flowcytometric analysis of W and AW leaves of *L. discolor* grown in soil; Figure S8. Aquaponic water system (AWS); Figure S9. Protein content in *L. discolor* leaves grown in AWS in comparison with soil-grown control; Table S1. Oligonucleotide primers used for amplification of DNA barcode regions; Table S2. Sequences information for DNA barcoding of *L. discolor*.
