# Supplementary material for: Jewel Orchid’s Biology and Physiological Response to Aquaponic Water as a Potential Fertilizer
Source: Plants (Basel). 2022 Nov 21;11(22):3181. doi: 10.3390/plants11223181 (PMC9699339; doi:10.3390/plants11223181)
Supplement: Supplementary file 1 [file plants-11-03181-s001.zip › plants-2018408-supplementary.pdf]

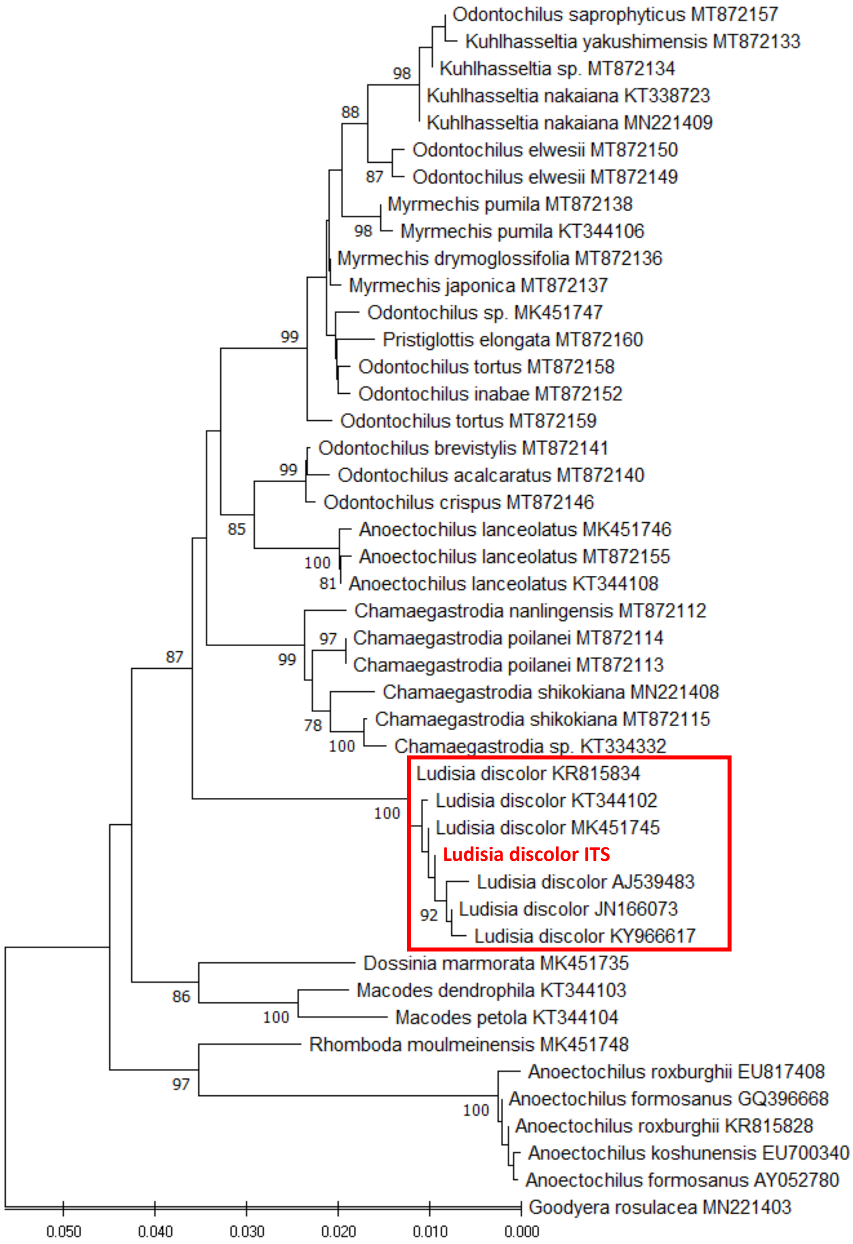

Figure S1. Phylogenetic tree of *Ludisia* sp. for the ITS DNA barcoding marker. In frame are highlighted the available *L. discolor* sequences in the database including the ITS sequences from this study. The percentage of replicate trees in which the associated taxa clustered together are shown next to the branches [12]. The tree is drawn to scale, with branch lengths in the same units as those of the evolutionary distances used to infer the phylogenetic tree. The evolutionary distances were computed using Neighbor-Joining method and Kimura 2-parameter model [13] and are in the units of the number of base substitutions per site. This analysis involved 45 nucleotide sequences. All ambiguous positions were removed for each sequence pair (pairwise deletion option).

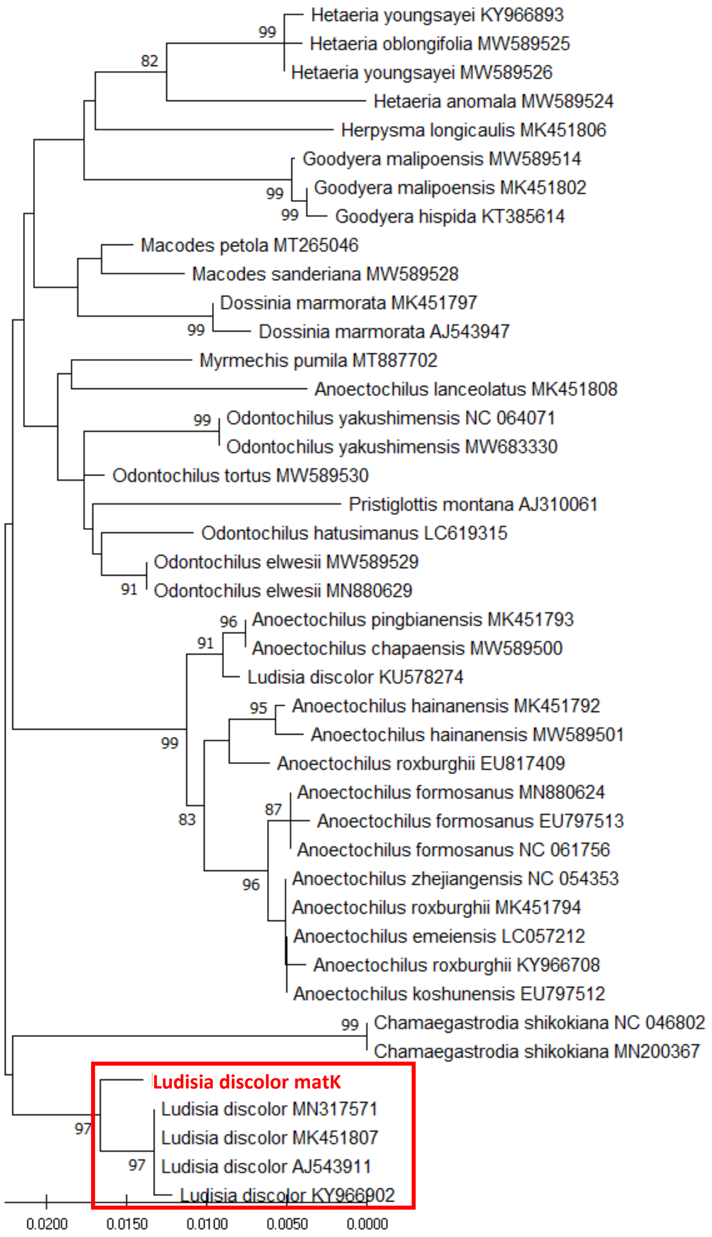

Figure S2. Phylogenetic tree of *Ludisia* sp. for the matK DNA barcoding marker. In frame are highlighted the available *L. discolor* sequences in the database including the matK sequences from this study. The percentage of replicate trees in which the associated taxa clustered together are shown next to the branches [12]. The tree is drawn to scale, with branch lengths in the same units as those of the evolutionary distances used to infer the phylogenetic tree. The evolutionary distances were computed using Neighbor-Joining method and Kimura 2-parameter model [13], and are in the units of the number of base substitutions per site. This analysis involved 42 nucleotide sequences for matK. All ambiguous positions were removed for each sequence pair (pairwise deletion option).

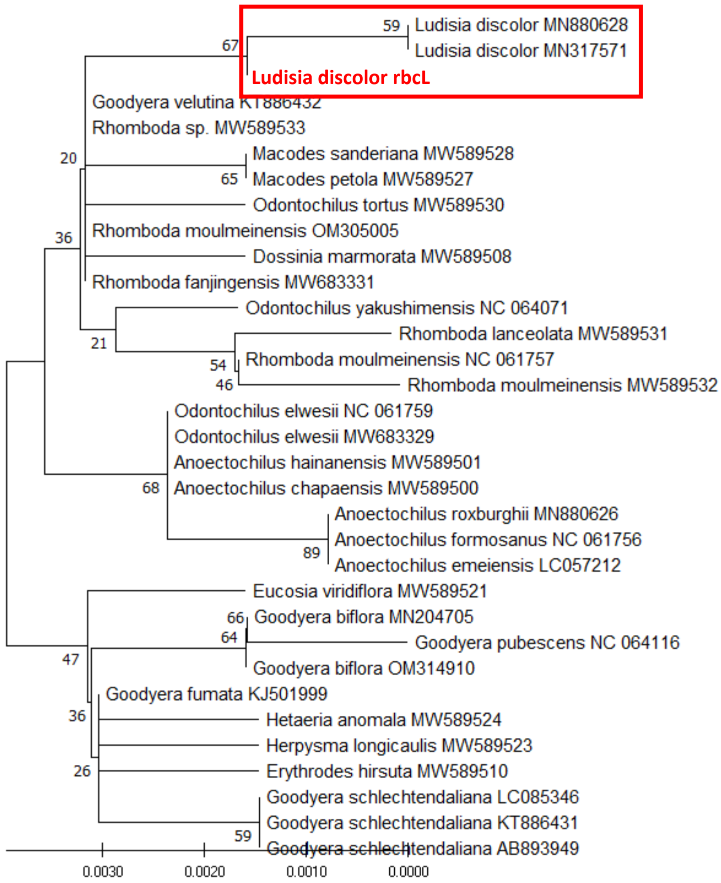

Figure S3. Phylogenetic tree of *Ludisia* sp. for the rbcL DNA barcoding marker. In frame are highlighted the available *L. discolor* sequences in the database including the rbcL sequences from this study. The percentage of replicate trees in which the associated taxa clustered together are shown next to the branches [12]. The tree is drawn to scale, with branch lengths in the same units as those of the evolutionary distances used to infer the phylogenetic tree. The evolutionary distances were computed using Neighbor-Joining method and Jukes-Cantor method [14] for rbcL and are in the units of the number of base substitutions per site. This analysis involved 33 nucleotide sequences. All ambiguous positions were removed for each sequence pair (pairwise deletion option).

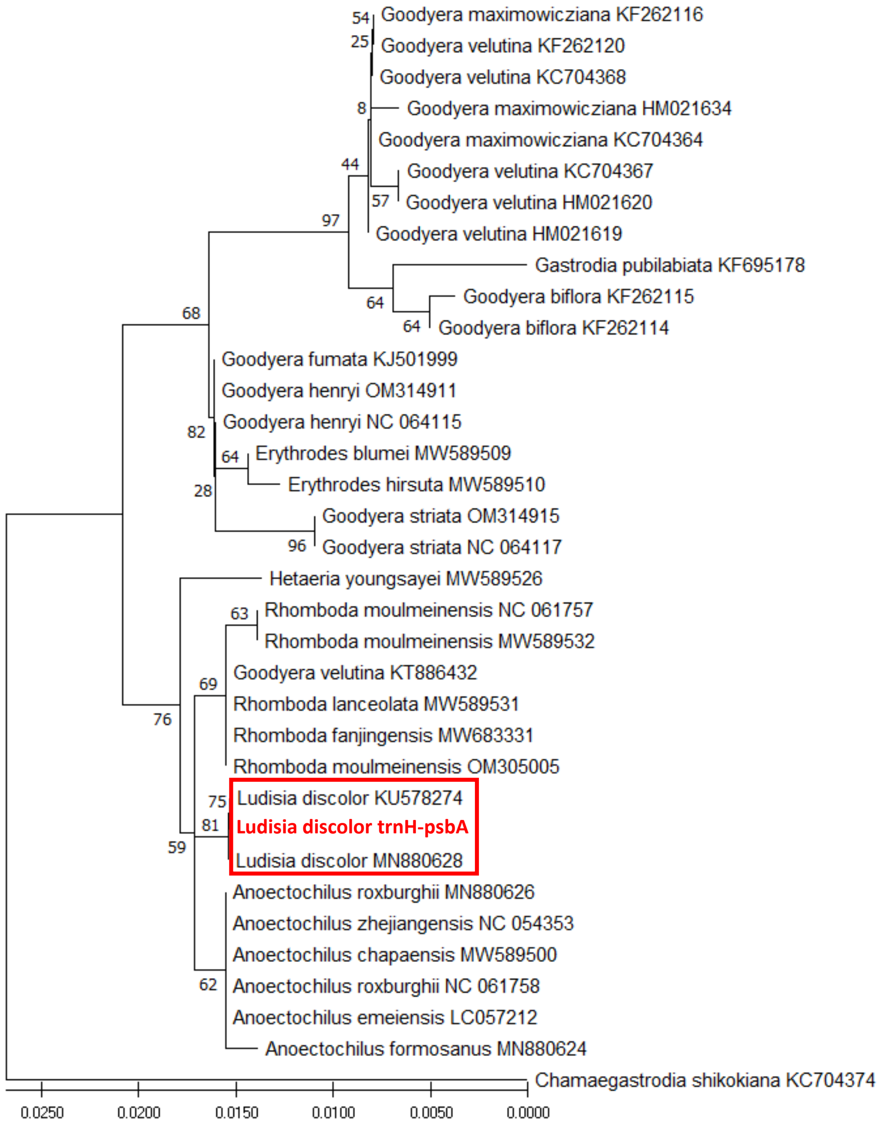

Figure S4. Phylogenetic tree of *Ludisia* sp. for the trnH-psbA DNA barcoding marker. In frame are highlighted the available *L. discolor* sequences in the database including the trnH-psbA sequences from this study. The percentage of replicate trees in which the associated taxa clustered together are shown next to the branches [12]. The tree is drawn to scale, with branch lengths in the same units as those of the evolutionary distances used to infer the phylogenetic tree. The evolutionary distances were computed using Neighbor-Joining method and Tamura 3-parameter model [15] and are in the units of the number of base substitutions per site. This analysis involved 35 nucleotide sequences. All ambiguous positions were removed for each sequence pair (pairwise deletion option).

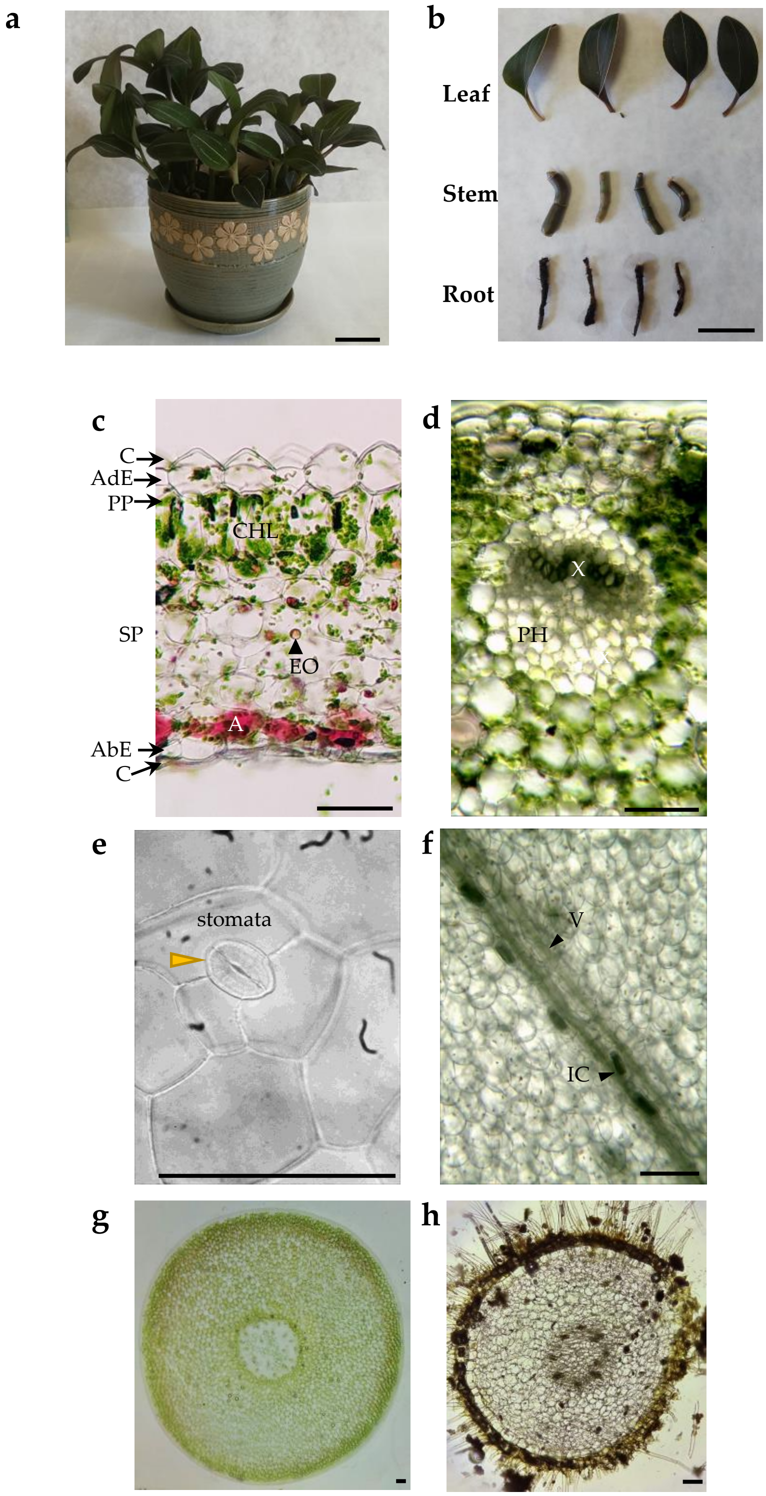

Figure S5. Anatomical overview of vegetative organs of *L. discolor* grown in soil. (a) Pot-grown plant; (b) Morphology of the vegetative organs studied; (c) Leaf cross section; C – cuticle; AdE – adaxial epidermis; PP – palisade parenchyma; SP – spongy parenchyma; AbE – abaxial epidermis. Marked are CHL – chloroplasts; A – anthocyanins; EO – essential oil drop. (d) Leaf cross section at vascular bundle; PH – phloem; X – xylem. (e) Abaxial epidermis – arrow indicates anisocytic type of stomata. (f) Idioblast cells (IC) and vasculature (V). (g) Stem cross section. (h) Root cross section. Scale bar 10 μm.

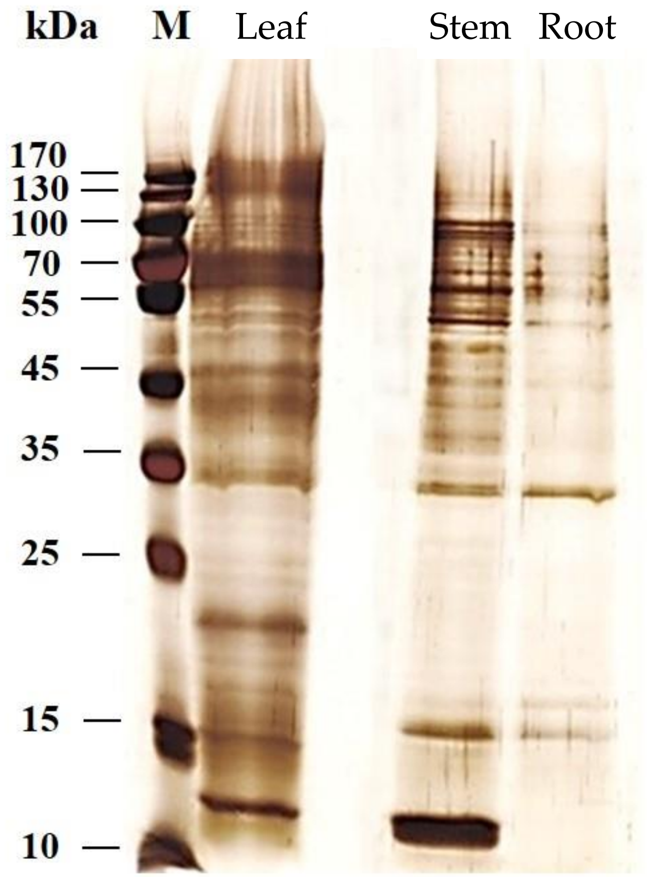

Figure S6. SDS PAGE protein profiles of *L. discolor* leaf, stem and root. Equal amount of extract was loaded on to each line.

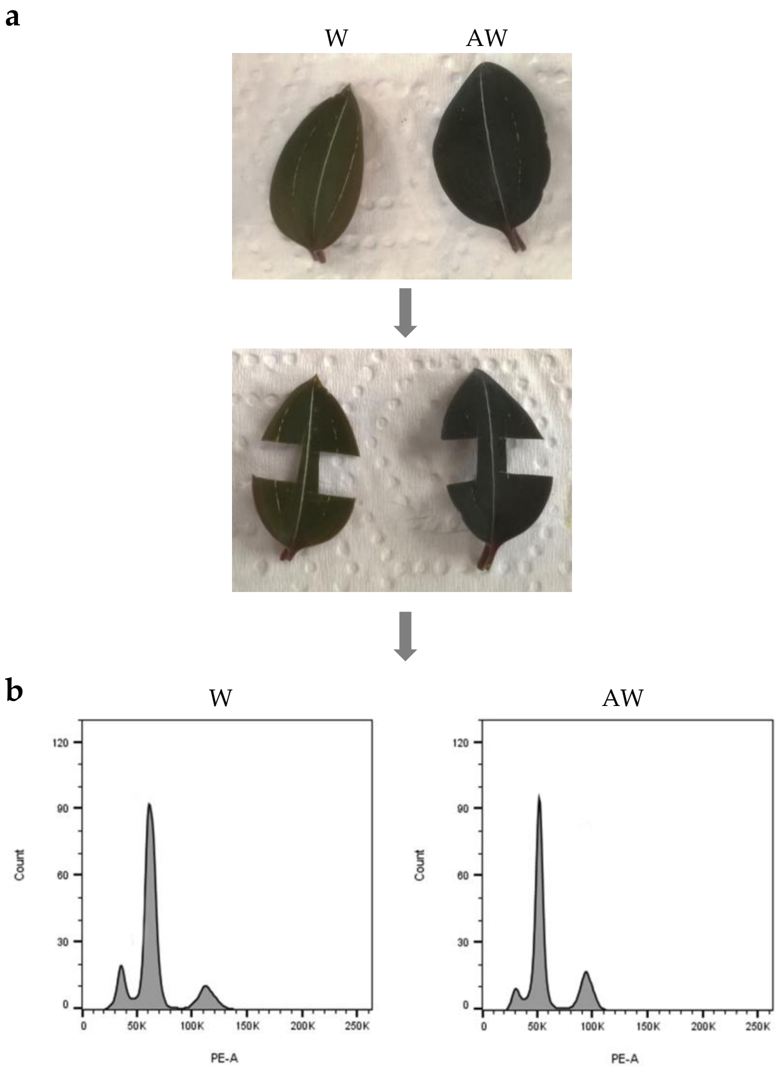

Figure S7. Flowcytometric analysis of W and AW leaves of *L. discolor* grown in soil. (a) Leaf samples for analysis. The cut leaf region taken for analysis is shown. (b) Flowcytometric data. W – water; AW – aquaponics water.

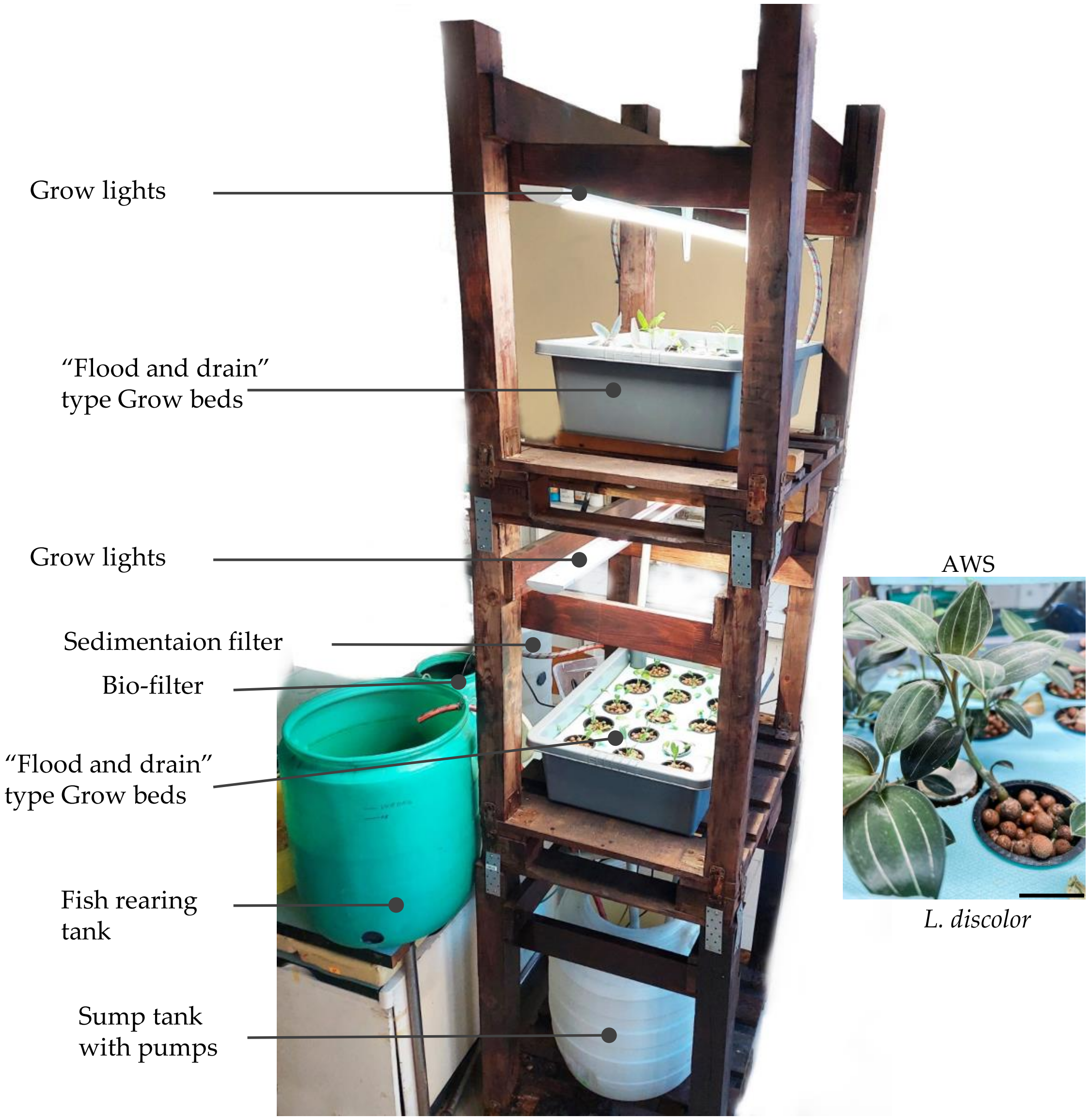

Figure S8. Aquaponic water system (AWS). The AWS has vertical aquaponics set-up, using a Media Bed system based on the model of "Info-graphic of an Aquaponic system made by Els Engel“. Adapted from [37]. Zoomed view of *L. discolor* in AWS.

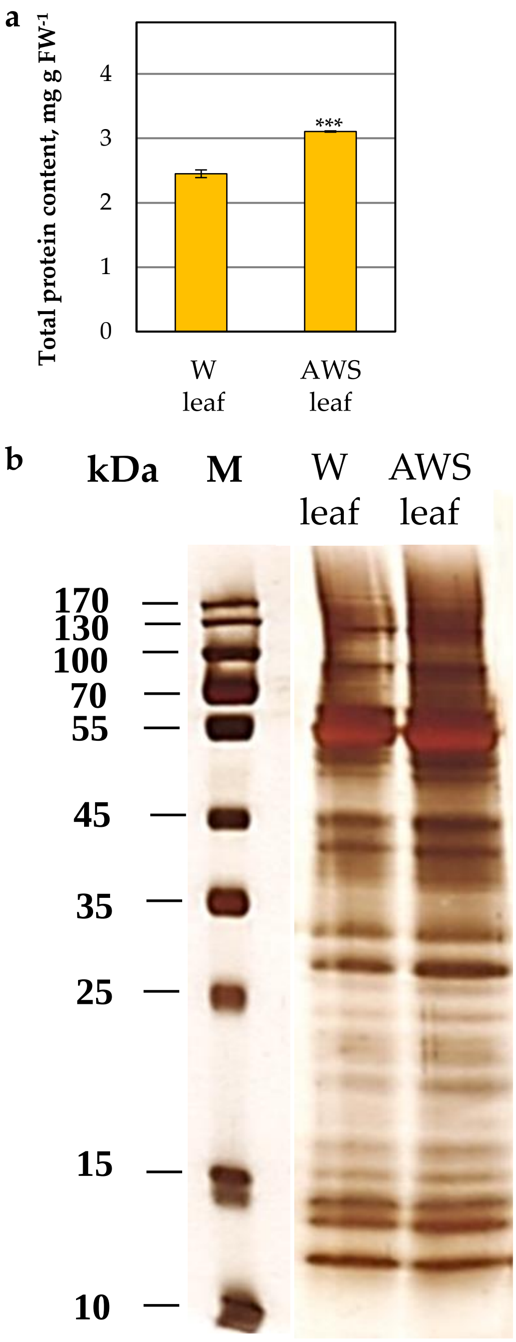

Figure S9. Protein content in *L. discolor* leaves grown in AWS in comparison with soil-grown control. (a) Total protein. (b) SDS-PAGE protein profile. Equal amount of extract was loaded on to each line. Protein ladder (M) indicates the approximate band sizes.

Table S1. Oligonucleotide primers used for amplification of DNA barcode regions.

Barcode Region	Primers	Primer Sequences 5′-3′	PCR Conditions	Reference
**ITS**	AB101	ACGAATTCATGGTCCGGTGAAGTGTTCG	94°C 5 min<br>94°C 30 s<br>58°C 1 min<br>72°C 1 min, 35x<br>72°C 5 min	[38]
	AB102	GAAACGGTCTCTCCAACGCAT		
rbcL	rbcLa-F	ATGTCACCACAAACAGAGACTAAAGC	94°C 4 min<br>94°C 30 s<br>55°C 30 s<br>72°C 1 min, 35x<br>72°C 10 min	[39]
	rbcLajf634R	GAAACGGTCTCTCCAACGCAT		[40]
matK	matK-KIM1R-f	ACCCAGTCCATCTGGAAATCTTGGTTC	94°C 4 min<br>94°C 30 s<br>51°C 60 s<br>72°C 60 s, 40x<br>72°C 4 min	[41]
	matK-KIM3F-r	CGTACAGTACTTTTGTGTTTACGAG		
trnH-psbA	psbA-trnH	CGCGCATGGTGGATTCACAATCC	94°C 4 min<br>94°C 30 s<br>55°C 30 s<br>72°C 1 min, 35x<br>72°C 7 min	[42]
	psbA-3F	GTTATGCATGAACGTAATGCTC		[43]

Table S2. Sequences information for DNA barcoding of *L. discolor*.

Barcode Region	Sequence 5′-3′ of *L. discolor*, sequence length (bp)
ITS<br>*~889 bp*	CATGGTCCGGTGAAGTGTTCGGATCGCTGTGACGTAGGTGGTTCGCTGCCCGCGACTCTGCGAGA<br>AGTCCATTGAACCTTATCATTTAGAGGAAGGAGAAGTCGTAACAAGGTTTCCGTAGGTGAACCTG<br>CGGAAGGATCATTGTCGAGACCCTAAAGAGGATTGGATGACTTTGGATAACACGTGAACAATTG<br>ACGACGATTGTCGTCTATAAGCACCATCCATCTATTGTCATTTCTTGATCGAAGTTACAATGAAAA<br>GATGGAAGGAAAACAACTCGGGCGCAGTTATGTGCCAAGGAAGTATGCTGCATAGGCATCGATG<br>GCTATTCGGCAAAGCCTGGCGTGCTTTGCGGAGTGCTGTTGTTGCTTCTTAAGTATTGTATGACTCT<br>CGGCAATGGATATCTTGGCTCTTGCATCGATGAAGAGCGCAGCGAAATGCGATACGTGGTGTGAA<br>TTGCAGAATCCCGTGAACCATCAAATCTTTGAACGCAAGTTGCGCCCGAGGCCATTTGGCTAAGG<br>GCACGTCCGCCTGGGCGTCAAGCATTACATCGCTTCATTCGACACCGATTGCCCAATTTTGTGCGG<br>TGGTGCCGATTCGAATGCGGAGAGTGGCCCTTCGCGCGTACTTGTGCGACGGGTTGAAGAACAGT<br>TTGCTTTCCTCTGGCCATGTTTTGATAAAGGGGTGGTGTATGCTGCCATTTGGCCCACGCTATCATC<br>TCATTGTCTTGAGGAAAATATCTGTACACATTCCTGGCTGTTCACCCGATATTGTCGTAGGTGGCG<br>CCCTGAAATGCGACCCCAGGTTGGGCGGGATGACCCGCTGAGTTTAAGCATATCAATAAGCGGA<br>GGAGAAGAAACTTACAAGGATTCCCTTAGTAACGGCGAGC
rbcL<br>*635 bp*	AAACAGAGACTAAAGCAAGCGTTGGATTTAAAGCTGGTGTTAAAGATTACAAGTTGACTTATTAT<br>ACTCCTGACTACGAAACCAAAAGTACTGATATCTTGGCAGCATTCCGAGTAACTCCTCAACCGGG<br>AGTTCCGCCTGAAGAAGCGGGCGCTGCGGTAGCAGCCGAATCTTCTACTGGTACATGGACAACTG<br>TGTGGACTGATGGACTTACCAGTCTTGATCGTTACAAAGGACGATGCTACCACATTGAGCCCGTT<br>GTTGGGGAGGAAAATCAATATATTGCTTATGTAGCTTATCCTTTAGACCTTTTTGAAGAAGGTTCT<br>GTTACTAACATGTTTACTTCCATTGTGGGTAATGTTTTTGGTTTCAAAGCCCTGCGAGCTCTACGTC<br>TGGAAGATCTGCGAATTCCCCCTTCTTATTCCAAAACTTTCCAAGGCCCGCCTCATGGCATCCAAG<br>TTGAAAGAGATAAATTGAACAAGTATGGTCGTCCCCTATTGGGATGTACTATTAAACCAAAATTG<br>GGATTATCCGCAAAAAACTACGGTCGAGCGGTTTATGAATGTTTACGGGGTGGACTTGATTTTACT<br>AAGGATGATGAAAACGTGAACTCACAACCATTTATGCGTTGGAGG
matK<br>*825 bp*	TGGAATCTTGGTTCAAATGCTTCAATGCTGGATCAAAGATGTTCCTTCTTTGCATTTGTTGCGATTT<br>ATTTTCCACGAATATTATAATTTGAAGAGTATCATTACTTCAAAGAAATCCATTCACGTTTTTTCAA<br>AAAAAAAGAAAAGATTTTTTTGGCTCCTACATAATTTTTATGTATATGAATGCGAATATCTTTTTCT<br>TTTTCTTCGTAAAAAGTCTTCTTATTTACGATCAACATCTTTTGGAGTCTTTATTGAGCGAACACTTT<br>TTCATGTAAAAATGGAATCTATTTTAGTAGTGTATTTTAATTCTTTTCAGAGGATTCTCTGGTTCCTC<br>AAAGATCCTTTCATACATTATGTTCGATATCAAGGAAAAGTAATTCTGGCTTCAAAGGGAACTCTT<br>ATTCTGATGAAGAAATGGAATTTTCATGTTGTGAATTTTTGGCAATTTTATTTTCACTTTTGGTCTCA<br>ACCTTATAGGATCCATATAAAGCAATTACCCAACTATTCCTTCTCTTTTCTGGGGTATTTTTTAAGT<br>GTACAAAAAAAAACTTTGGTAGTAAGAAATCAAATGCTAGAGAATTCCTTTCTAATAAATACTCT<br>GACTAAGAAATTAGATACCATAGCCCCAGTTATTTCTCTTATTGGATCATTGTCGAAAGCTCAATT<br>TTGTACTATATCGGGTCATCCTATTAGTAAACCAATTTGGACCGATTTATCGGATTCTGATATTATT<br>GATCGATTTTGTCGAAAATGTAGAAATCTTTGTCGTTATCACAGCGGATCCTCAAAAAAAAAAGT<br>TTTGTATCGTATAAAATATATACT
trnH-psbA<br>*723 bp*	AAAAARGTAAAAAAAAAAAAAAAAAAAAAGGAGTAATCGGCCGTGACACGTTCACTAAAAAA<br>AAATCCTTTTGTAGCTAATCATTTATCGGTAAAAATTGAAAAACTCAACATGAGGGAGGAGAAA<br>GAAATAATAGTGACTTGGTCTAGGGCATCTACCATTATACCCACAATGATTGGCCATACAATCGC<br>TATTCATAATGGAAAGGAACATTTACCTATTTATATAACAGATCGTATGGTCGGTCACAAATTGG<br>GAGAATTCGCACCTACTCTCACTTTCGTGAGACATGCGAGAAATGATAATAAATCTCGTCGTTAG<br>TCGTTCTACTAAGTATTCATGTGAAAAGTCTTATCTTAATAGTATTTCAACTTAAGAGTCTTTATCT<br>TATAGTAAGAGTATAGGTATAGTCTTTTATAGTATACTAATATACTAAGACTTAGATTTTTCTTACT<br>TATCTTATACTATACTTCACCTAGGCACTTATCATTCATTGGCGGGGGAGAACTTTCTTTTATGATA<br>AAGAACGAAAATTCGGAGAAATCGGATAAAGAAGCAAAAGTGTTAGCTCAACATATACATATGT<br>CTGTTTTCAAAGCACGAAGAGTAATAGCAATCCCCAATATCCAACATATTGGATATTGGGGATTG<br>CTACCTTAAAGAATTCATCTACATAGAAAATAAATAAATACAAAAGTATTATCCATTTGTAGATG<br>GAGCTCC
